# Construction of an AI-2 quorum sensing induced heterologous protein expression system in *Escherichia coli*

**DOI:** 10.7717/peerj.12497

**Published:** 2021-11-16

**Authors:** Fei Shang, Hui Wang, Dan Zhang, Wenhui Wang, Jiangliu Yu, Ting Xue

**Affiliations:** Anhui Agricultural University, School of Life Sciences, Hefei, Anhui, China

**Keywords:** AI-2 quorum sensing, Protein expression, Auto-induction, *Escherichia coli*

## Abstract

**Background:**

The pET expression system based on T7 promoter which is induced by isopropyl-β-D-1-thiogalactopyranoside (IPTG) is by far the most commonly used system for production of heterogeneous proteins in *Escherichia coli*. However, this system was limited by obvious drawbacks including the host toxicity and metabolic burden imposed by the presence of IPTG.

**Methods:**

In this study, we incorporated the autoinducer-2 (AI-2) quorum sensing system to realize autoinduction of the pET expression system. The autoinduction expression vector pXWZ1 was constructed by inserting the *lsr* promoter regions into the pET28a(+) vector. The expression efficiency of the reporter genes *gfpuv* and *lacZ* by the pXWZ1 and pET28a(+) vectors were compared.

**Results:**

The results showed that the expression levels of the both report genes in the cells transformed with pXWZ1 without any addition of exogenous inducer were higher than that transformed with pET28a(+) vectors by the induction of IPTG.

**Conclusion:**

This new auto-induction system will exclude the limitations of the IPTG induction including toxic to host and increasing formation of inclusion body and will become a more economical and convenient tool for recombinant protein expression.

## Introduction

Among the many microbial hosts used for recombinant protein expression, *Escherichia coli* is the most preferable one because of its well-studied genetics, low culturing expenses, and the characteristics of rapid growth and high production yield (Huang, Lin & Yang, 2012; Jia & Jeon, 2016; Makrides, 1996; Rosano & Ceccarelli, 2014). As far as we know, a large number of cloning plasmids and mutant *E. coli* strains have been used in laboratory research and industrial production. And among them, the system known as the pET vectors in combination with the *E. coli* strain BL21(DE3) has gained increasing popularity (Gay et al., 2014; Jia & Jeon, 2016; Rosenberg et al., 1987; Wurm et al., 2016).

The pET vector was derived from the medium copy number plasmid pBR322, and has developed as a series of variants which are widely used for heterologous protein expression. In the pET series vectors, target genes are inserted downstream of the T7 promoter, and the T7 phage RNA polymerase recognizes the promoter and initiates transcription of target gene. The expression of the T7 RNA polymerase gene was induced by isopropyl-β-D-thiogalactopyranoside (IPTG) (Sorensen & Mortensen, 2005; Studier & Moffatt, 1986; Studier et al., 1990). Although this system realizes high level of protein expression, which accounts for about 40–50% of the total cell protein, there are also some obvious drawbacks preventing it from being a common choice for industrial applications. These disadvantages mainly arise from the metabolic burden and toxicity of IPTG on *E. coli* and rapid over-expressions of proteins that lead to increasing formation of inclusion bodies (IBs) (Dvorak et al., 2015; Gatti-Lafranconi et al., 2011; Glick, 1995; Haddadin & Harcum, 2005). In recent years, certain strategies have been addressed to overcome these limitations. For example, Dvorak et al. (2015) suggested tuning down the transcription rate of the recombinant protein by decreasing the using amounts of IPTG. Several other studies have proposed applying lactose as inducer instead of IPTG to enhance correct protein folding and increase cell fitness (Neubauer et al., 1992; Ramchuran, Holst & Karlsson, 2005). However, the above problems cannot be absolutely solved. Moreover, in view of the expensive cost and toxicity of IPTG, researchers also attempted to explore novel promoters which are not induced by IPTG. Several studies have utilized bacterial quorum sensing (QS) system to enhance the yield of recombinant proteins (March & Bentley, 2004; Studier, 2014).

**Figure 1:**
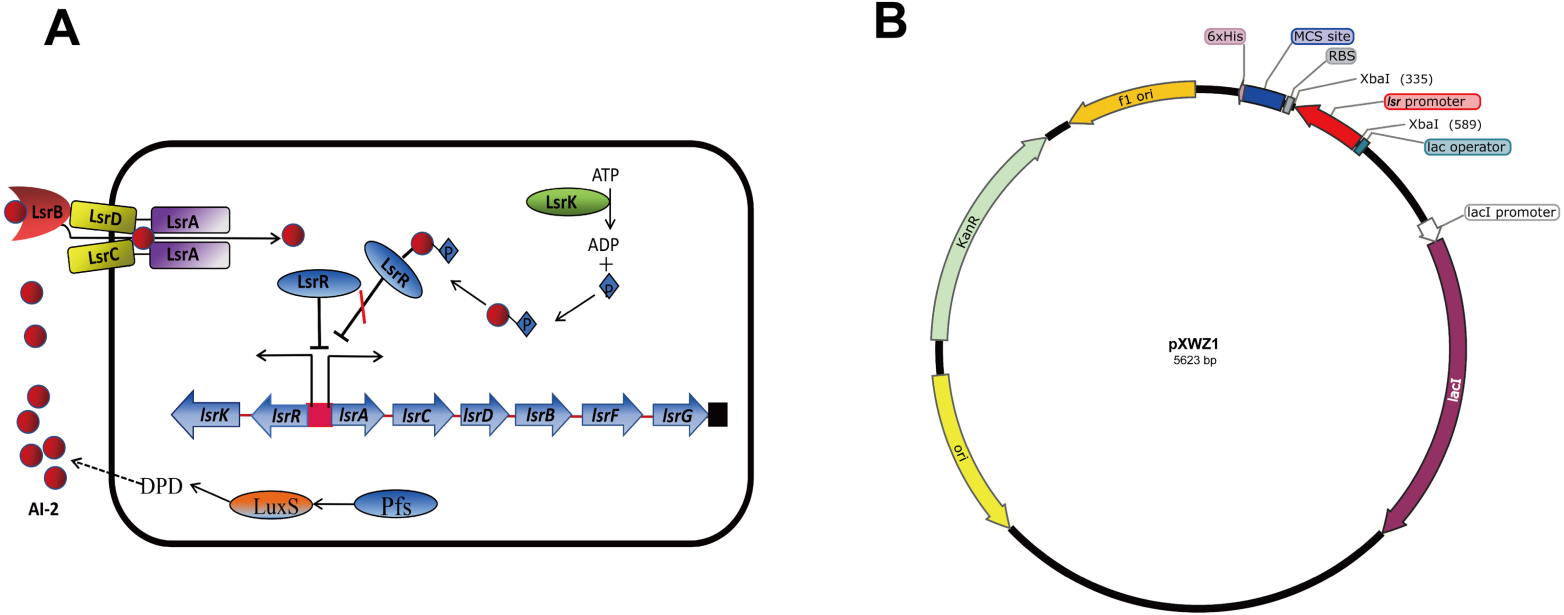
(A) Model for regulation, transportation, and modification of AI-2 by the LsrR proteins in *E. coli*. (B) Sketch map of the constructed pXWZ1 vector.

QS is a process that bacteria utilize self-produced and released molecules as cell-to-cell communicating signals to control gene expression. When the concentration of the external signal molecules referred as autoinducers reaches a threshold, bacteria modify the gene expression profile to function as multicellular organisms (Bassler, 1999; Waters & Bassler, 2005). Autoinducer-2 (AI-2) QS system is widely conserved among Gram-negative and Gram-positive bacteria and has been considered to be used for interspecies communication (Federle & Bassler, 2003; Vendeville et al., 2005). In *E.coli,* proteins associated with detection and transportation of AI-2 are encoded by the *lsrACDBFG* operon, and *lsrRK,* which are divergently transcribed from the *lsr* operon (Fig. 1A). The genes *lsrACDB* are responsible for the production of ATP-binding cassette transporter components which are involved in AI-2 uptake; the genes *lsrFG* are considered to be associated with the modification of AI-2 following internalization. The *lsr* operon is regulated by *lsrR*, which encodes the repressor of the *lsr* operon, and *lsrK*, which encoded a kinase responsible for converting AI-2 to phospho-AI-2. LsrR represses the transcription of *lsr* operon and itself by binding to their promoter regions, and the repression can be relieved in the presence of phospho-AI-2 (Taga, Miller & Bassler, 2003; Xavier & Bassler, 2005a; Xavier & Bassler, 2005b; Xue et al., 2009). β-galactosylase is the most common reporter gene used for studying gene expression in bacteriology. There are many advantages of it including the ability to function in a wide range of bacteria enzyme analysis, availability of substrates for genetic screening, and the various tools developed to construct *lacZ* gene fusions. Green fluorescent protein (GFP) is another transcriptional reporter gene with a broad host range, which is complementary to β-galactosidase in many aspects (Goulian & Van der Woude, 2006).

In this study, we attempted to modify the pET28a(+) vector by incorporating the AI-2 auto-induction system. We constructed pXWZ1 vector by inserting the *lsr* promoter region into the pET28a(+) vector. The results indicated that the expression of the target gene in pXWZ1 vector was not relying on any addition of exogenous inducer. Furthermore, the expression levels of the reporter genes (*gfpuv* and *lacZ*) by the induction of IPTG and AI-2/LsrR system were compared between the pET28a(+) vector and pXWZ1 vector.

## Materials and Methods

### Bacterial strains and media

The bacterial strains and plasmids used in this study are listed in Table 1. All *E. coli* strains were cultured in lysogeny broth (LB) at 37 °C with aeration. When necessary, media were supplemented with antibiotics at the following concentrations (mg/L); ampicillin (Amp), 150; kanamycin (Kan), 50.

**Table 1:** The bacterial strains and plasmids used in this study.

Strain or plasmid	Relevant genotype	Reference or source
Strains		
*E. coli*		
DH5α	Clone host strain, *supE44*Δ*lacU169*(ϕ80 *lacZ*ΔM15) *hsdR17 recA1 endA1 gyrA96 thi-1 relA1*	Invitrogen
BL21	Expression strain, F^−^*ompT hsdS*(r_B_^−^ m_B_^−^) *gal dcm* (DE3)	Invitrogen
MG1655	F^−^λ^−^*rph*-*1*	Blattner et al. (1997)
DH5α/pGFPuv	DH5α with the plasmid pGFPuv, Cm^r^^a^	Laboratory stock
DH5α/pXWZ1	DH5α with the plasmid pXWZ1, Kan^r^^a^	This study
BL21/pET28a-GFPuv	BL21 with the plasmid pET28a-GFPuv, Kan^r^	This study
BL21/pXWZ1-GFPuv	BL21 with the plasmid pXWZ1-GFPuv, Kan^r^	This study
BL21/pET28a-LacZ	BL21 with the plasmid pET28a-LacZ, Kan^r^	This study
BL21/pXWZ1-LacZ	BL21 with the plasmid pXWZ1-LacZ, Kan^r^	This study
Plasmids		
pET28a(+)	Expression vector, Kan^r^	Novagen
pGFPuv	The vector with *GFPuv* gene, Cm^r^	Laboratory stock
pXWZ1	The expression vector containing the *lsrA* promoter, Kan^r^	This study
pET28a-GFPuv	pET28a(+) with *GFPuv* gene, Kan^r^	This study
pXWZ1-GFPuv	pXWZ1 with *GFPuv* gene, Kan^r^	This study
pET28a-LacZ	pET28a(+) with *lacZ* gene, Kan^r^	This study
pXWZ1-LacZ	pXWZ1 with *lacZ* gene, Kan^r^	This study

**Notes.**

a Cm^r^, chloramphenicol-resistant; Kan^r^, kanamycin-resistant.

### Plasmid construction

The *lsr* promoter region was amplified from the genome DNA of the *E.coli* strain MG1655 by performing standard PCR experiments according to the instructions of Ezup Column Bacteria Genomic DNA Purification Kit (Sangon Biotech , Shang Hai) using primers *plsr*-Xba I-f and *plsr*-Xba I-r. The pET28a(+) vector was extracted from *E.coli* strain DH5α by using the SanPrep Column Plasmid Mini-Preps Kit (Sangon Biotech, Shang Hai), digested with the restriction endonuclease Xba I, and then ligated with the *lsr* promoter fragment which was also digested by Xba I to finally obtain the pXWZ1 expression vector. Green fluorescent protein encoding-gene *gfpuv* was amplified from the vector pGFPuv using primes *GFPuv*-EcoR I-f and *GFPuv*-Hind III-r. The *gfpuv* gene fragment, pET-28 and pXWZ1 were all digested with restriction endonuclease EcoR I-f and Hind III and ligated to create the vectors pET28a-GFPuv and pXWZ1-GFPuv. The β-galactosidase expression gene *lacZ* was amplified from the genome of *E.coli* strain MG1655 using primers *lacZ*-BamH I-f and *lacZ*-Hind III-r. To create the vectors pET28a-GFPuv and pXWZ1-GFPuv, the *lacZ* gene fragment, pET28a(+) and pXWZ1 were digested with restriction endonuclease BamH I and Hind III and ligated. All constructed vectors were transformed into the *E.coli* strain BL21(DE3), respectively. The primers used in this study are listed in Table 2.

**Table 2:** Oligonucleotide primers used in this study.

Primer name	Oligonucleotide (5′-′)^a^
*p-lsr*-Xba I-f:	GCTCTAGAAATTCATTCTTCACTTTGAA
*p-lsr*-Xba I-r:	GCTCTAGAATTTCCCCCGTTCAGTTTTG
GFPuv-EcoR I-f:	CCGGAATTCATGAGTAAAGGAGAAGAACT
GFPuv-Hind III-r:	CCCAAGCTTTTATTTGTATAGTTCATCCA
*lacZ*-BamH I-f:	CGCGGATCCATGACCATGATTACGGATTC
*lacZ*-Hind III-r:	CCCAAGCTTTTATTTTTGACACCAGACCA
T7-f:	TAATACGACTCACTATAGGG
T7-r:	TGCTAGTTATTGCTCAGCGG
check-*lacZ*-f	TACAGGGCGGCTTCGT
check-*lacZ*-r	GCGGGTCGCTTCACTTAC

**Notes.**

a The sequences with the underline refer to the restriction endonuclease recognition sites.

### Measurement of GFP fluorescence

The BL21(DE3) cells, which were transformed with pET28a-GFPuv and pXWZ1-GFPuv, respectively, were all grown overnight at 37 °C, and inoculated into 100 mL 50 mg/mL fresh LB medium with Kanamycin as a ratio of 1:100 v/v, and subsequently cultured at 37 °C with shaking at 150 rpm to an optical density (OD600 nm) of 0.3. The pET28a-GFPuv transformed group was added with IPTG to a final concentration of 0.5 mmoL/L. The cells were collected at 2 h intervals, and rinsed twice with 1% NaCl. The GFPuv fluorescence intensity was detected by using an inverted fluorescence microscope (Nikon, Tokyo, Japan). In addition, the fluorescence intensity value was measured by using a multi-function microplate reader SpectraMax M5 (Molecular DevicesCorporation, California, USA), and the whole cell determination of GFPuv was performed at the excitation wavelength of 395nm and the emission wavelength of 509nm. The LB medium was set as a negative control. All the experiments were repeated at least three times.

### β-galactosidase assay

The BL21(DE3) cells transformed with pET28a-GFPuv and pXWZ1-GFPuv were grown overnight and diluted 1:100 into 100 mL fresh LB medium with50 mg/mL Kanamycin, and cultured to an optical density (OD600 nm) of 0.3. The cells (0.005 mL) were harvested and resuspended in 1 ml of Z-buffer. After addition of 100 ul chloroform and 50 ul 0.1% SDS, the cells are vortexed and incubated at 30 °C for 5 min. The reaction was initiated by the addition of 0.2 mL of o-nitrophenyl-b-D-galactopyranoside (ONPG; 4 mg/ml) in Z-buffer and was ended with the addition of 0.5 mL 1 M Na_2_CO_3_. The enzymatic activity was monitored by reading the A420 of a UV/Vis spectrophotometer (Thermo Scientific, Pittsburgh, PA, USA). β-Galactosidase units are defined as (OD 420 × 1 000)/ (OD 600 × Volume (ml) × Time (min)). All assays are reported as the mean β-galactosidase activity of three independent cultures.

### SDS–polyacrylamide gel electrophoresis (SDS-PAGE) assay

To determine the ratio of the protein expressed by pET28 and pXWZ1 vector in the form of inclusion bodies, the QseB and GFPuv proteins were expressed and compared. The BL21(DE3) cells, which were transformed with pET28a-GFPuv and pXWZ1-GFPuv or pET28a-QseB and pXWZ1-QseB, respectively, were all grown overnight at 37 °C, and inoculated into 100 mL 50 µg/mL fresh LB medium with Kanamycin as a ratio of 1:100 v/v, and subsequently cultured at 37 °C with shaking at 150 rpm to an optical density (OD600 nm) of 0.3. The pET28a-QseB and pET28a-GFPuv transformed group was added with IPTG to a final concentration of 0.5 mmoL/L. Subsequently, the cells were collected after culturing for 12 h for ultrasonic cell fragmentation. The crushed cells were centrifuged at 12,000 rpm for 20 min to separate the supernatant and precipitate for the following SDS-PAGE.

### Statistical analysis

The data were analyzed using the statistical software Prism 8 (GraphPad Software Inc., La Jolla, CA) by a one-way ANVONA method; the test results were shown as mean ± SD. The paired *t*-test was used for statistical comparisons between groups. The level of statistical significance was set at a *P*-value of ≤ 0.05.

## Results

### Construction of the recombinant vectors

The *lsr* operon promoter region (−248 to −1 relative to the start codon of *lsrA*) was amplified from the genome of the *E.coli* strain MG1655. To construct the pXWZ1 expression vector, the *lsr* promoter was inserted into the pET28a(+) vector at the restriction endonuclease site of XbaI (Fig. 1B). The pET28a-GFPuv and pXWZ1- GFPuv expression vectors were generated by incorporating green fluorescent protein (GFP) encoding-gene *gfpuv* amplified from the vector pGFPuv into the pET28a(+) and pXWZ1 expression vectors, respectively. In addition, the vectors pET28a-LacZ and pXWZ1- LacZ were obtained by inserting the reporter gene *lacZ* amplified from the genome of *E.coli* strain MG1655 into the pET28a(+) and pXWZ1 expression vectors, respectively.

### Measurement of the GFP fluorescence to compare the expression efficiency of vectors pET28a-GFPuv and pXWZ1- GFPuv

**Figure 2:**
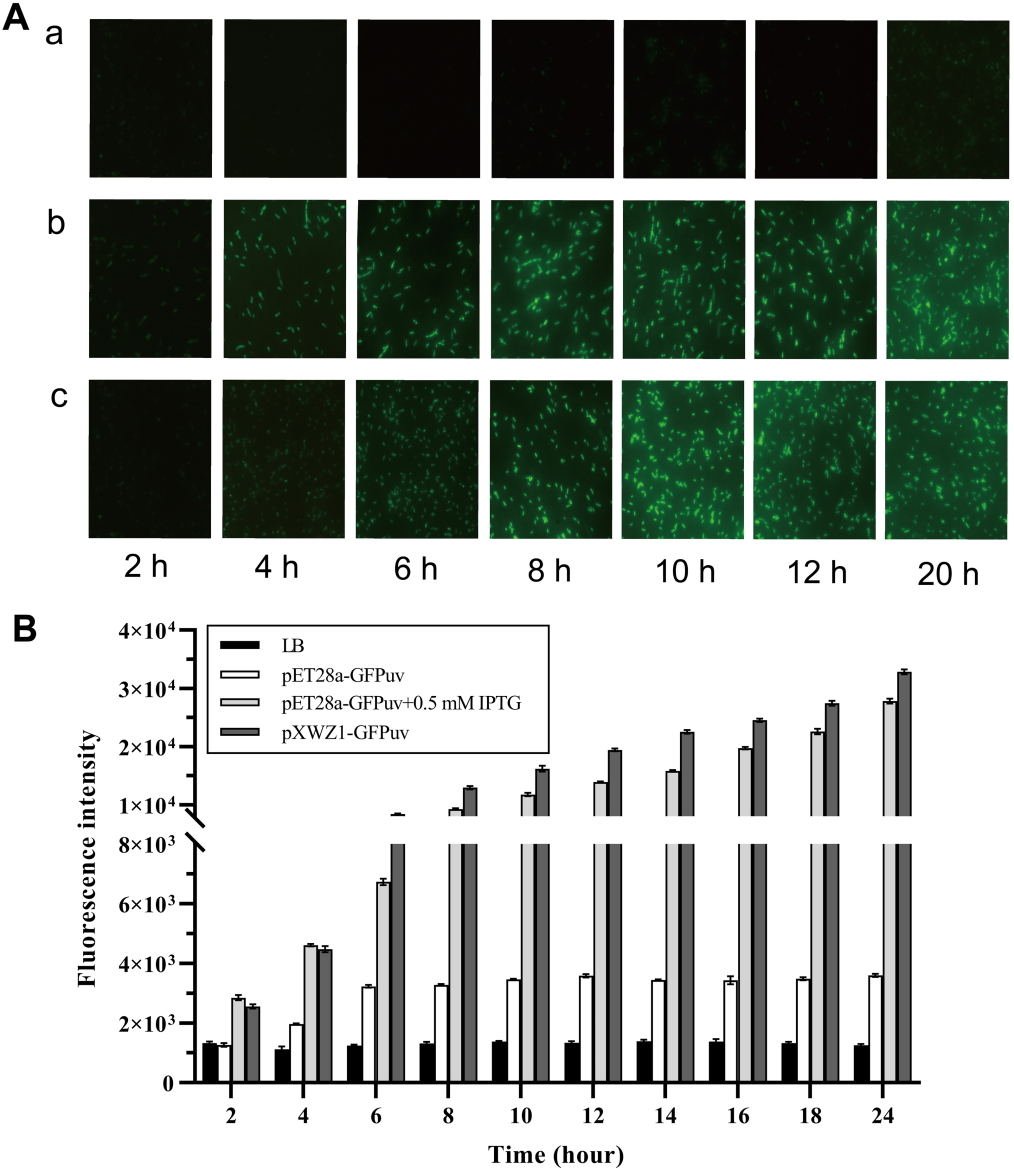
Measurement of the GFP expression levels. (A) Fluorescence microscope photographs of the *E. coli* BL21(DE3) cells transformed with pET28a-GFPuv(b) and pXWZ1-GFPuv(c) vectors, respectively (1,000× magnification). (B) The fluorescence intensity value was further measured by using a multi-function microplate reader SpectraMax M5. (1) only LB medium (2) cells transformed with pET28-GFPuv (3) cells transformed with pET28a-GFPuv in the presence of 0.5 mM IPTG (4) cells transformed with pXWZ1- GFPuv. The cultures were harvested at 2 h intervals. Error bars indicate standard deviations.

In order to evaluate the expression efficiency of the pXWZ1 expression vector, the expression levels of the reporter gene *gfpuv* were compared between vectors pET28-GFPuv and pXWZ1- GFPuv. The both vectors were transformed into the *E.coli* strain BL21(DE3), respectively. Since IPTG induction is essential for target gene expression in the pET28a(+) vector, the GFP expression were also measured with addition of IPTG in the medium cultured cells carrying the pET28a-GFPuv vector. Equal amounts of three groups of samples were taken at time intervals and were evaluated for GFPuv fluorescence. The GFPuv fluorescence was observed under a fluorescence microscope (1, 000 × magnification). The results showed that after 6 h, the GFP expression level in the pXWZ1-GFPuv transformed group were no less than that of the pET28a-GFPuv transformed group with the induction of IPTG (Fig. 2A). The fluorescence intensity value was further measured by using a multi-function microplate reader SpectraMax M5 (Molecular DevicesCorporation, California, USA), and the whole cell determination of GFPuv was performed at the excitation wavelength of 395nm and the emission wavelength of 509nm. As shown in Fig. 2B, at each time point after 4 h, the fluorescence intensity from the cells transformed with pXWZ1- GFPuv was higher than that from the IPTG-induced cells transformed with pET28a-GFPuv.

### Measurement of the β-galactosidase activity to compare the expression efficiency of vectors pET28a-LacZ and pXWZ1- LacZ

**Figure 3:**
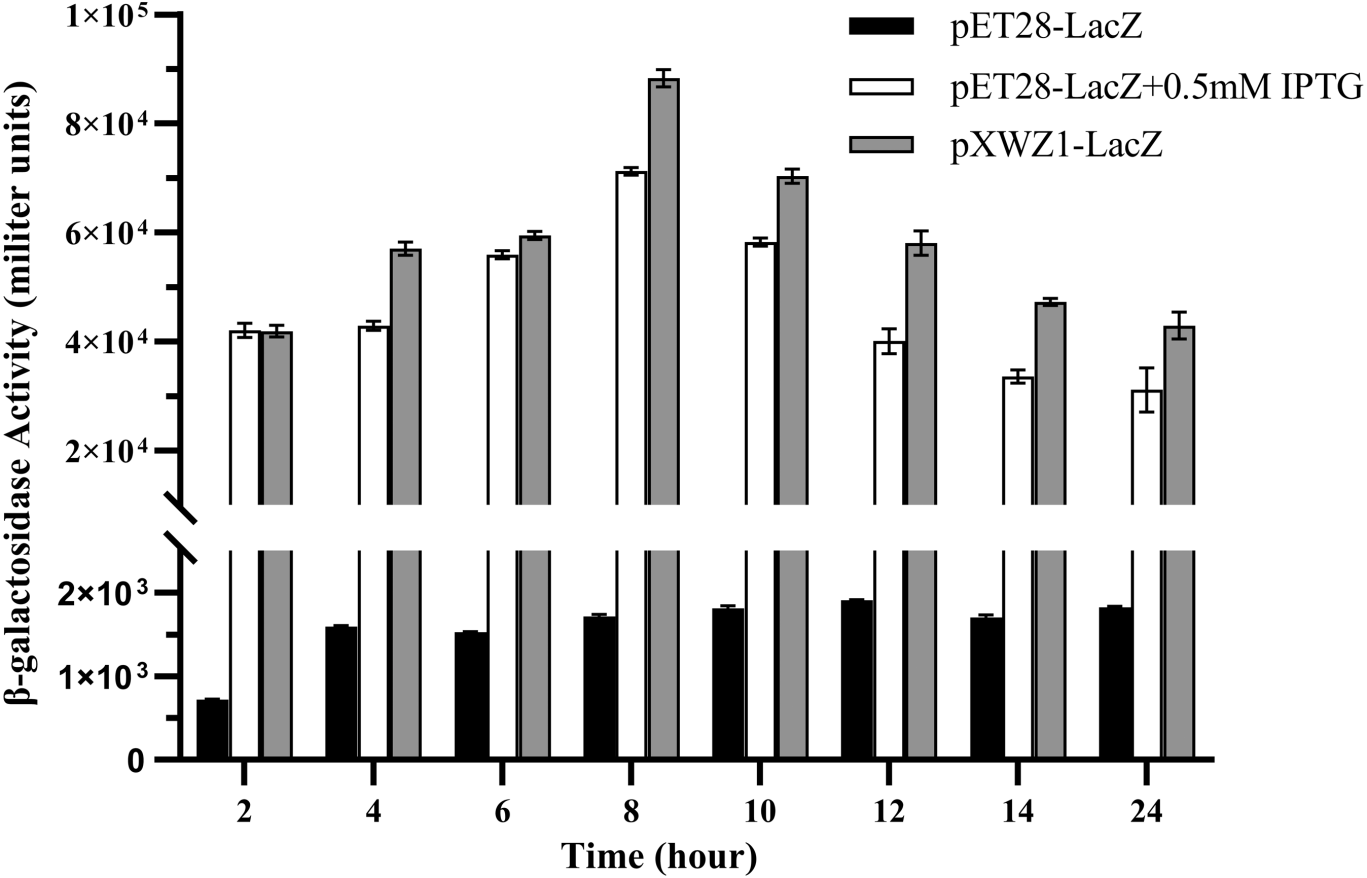
Measurement of the β-galactosidase activity. β-galactosidase activities of the *E. coli* B21(DE3) cells transformed with corresponding vectors were measured at 2 h intervals. (1) cells transformed with pET28a-LacZ (2) cells transformed with pET28a-LacZ in the presence of 0.5 mM IPTG (3) cells transformed with pXWZ1- LacZ. Error bars indicate standard deviations.

In order to further evaluate the expression efficiency of the pXWZ1 expression vector, the expression levels of another reporter gene *lacZ* were compared between vectors pET28a-LacZ and pXWZ1-LacZ. The both vectors were also transformed into the *E. coli* strain BL21(DE3), respectively. In the pET28a-LacZ transformed group, IPTG was added at the optimal concentration of 0.5 mmoL/L and at 0 mmoL/L as a negative control, respectively. The β-galactosidase activities were measured at time intervals in equal amounts of cells transformed with pET28a-LacZ and the cells transformed with the pXWZ1-LacZ vector, respectively. The results showed that at each time interval after 4 h, the β-galactosidase activity of the pXWZ1-LacZ transformed group was higher than that of the pET28a-LacZ transformed group with the induction of IPTG (Fig. 3).

### Detection of the difference between pET28(+) and pXWZ1 expressing the amount of inclusion body protein

**Figure 4:**
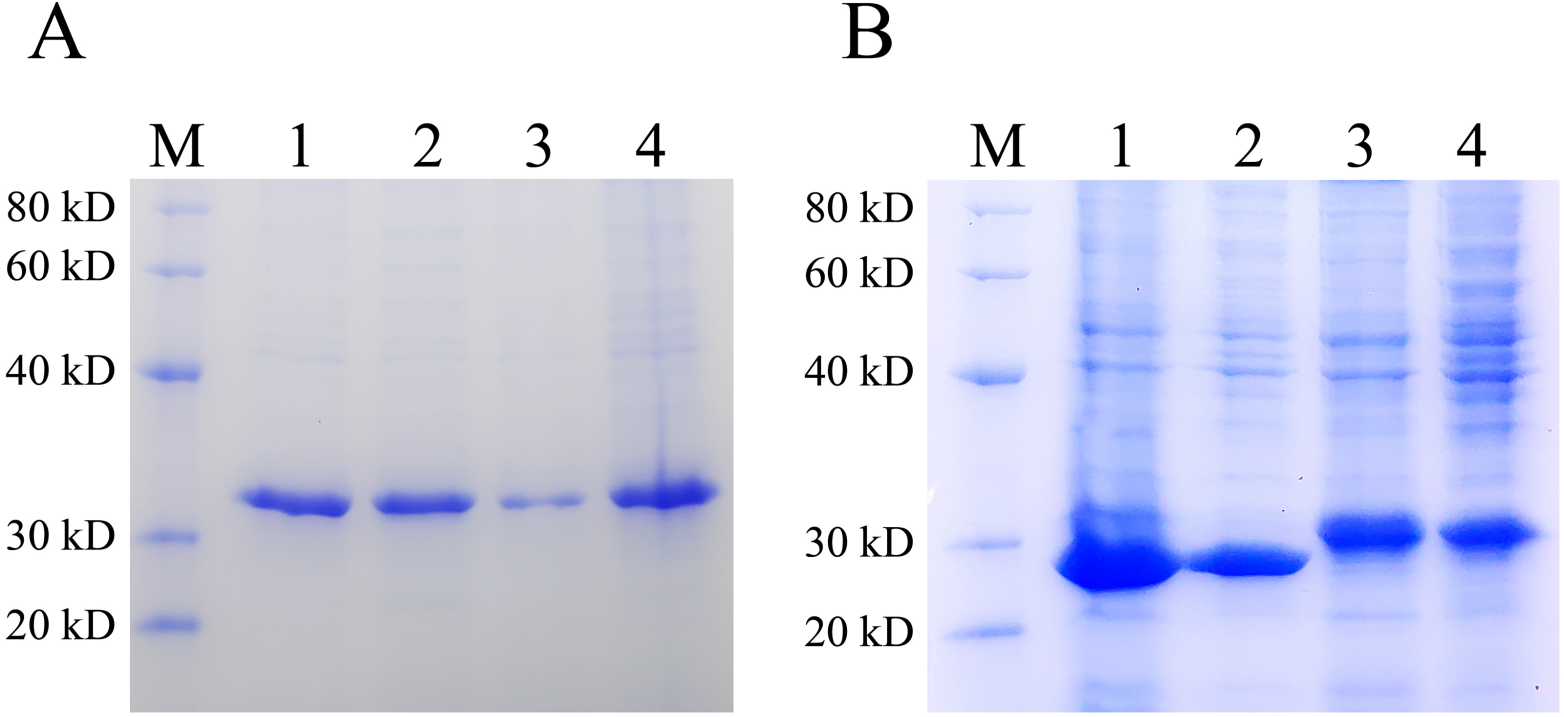
Detection of the difference between pET28(+) and pXWZ1 expressing the amount of inclusion body protein by SDS-PAGE. (A) A Coomassie-stained SDS-PAGE gel of the supernatant and precipitation of the crushed cells BL21(DE3)/pET28-GFPuv or BL21(DE3)/pXWZ1-GFPuv. M, protein marker; lane 1, the precipitation of crushed cells BL21(DE3)/pET28-GFPuv; lane 2, the supernatant of crushed cells BL21(DE3)/pET28-GFPuv; lane 3, the precipitation of crushed cells BL21(DE3)/pXWZ1-GFPuv; lane 4, the supernatant of crushed cells BL21(DE3)/pXWZ1-GFPuv. (B) A Coomassie-stained SDS-PAGE gel of the supernatant and precipitation of crushed cells BL21(DE3)/pET28-QseB or BL21(DE3)/pXWZ1-QseB. M, protein marker; lane 1, the precipitation of crushed cells BL21(DE3)/pET28-QseB; lane 2, the supernatant of crushed cells BL21(DE3)/pET28-QseB; lane 3, the precipitation of crushed cells BL21(DE3)/pXWZ1-QseB; lane 4, the supernatant of crushed cells BL21(DE3)/pXWZ1- QseB.

Inclusion bodies are high-density, insoluble protein particles wrapped by membranes formed when exogenous genes are expressed in prokaryotic cells, especially in *E. coli*. Proteins in inclusion bodies are aggregates in an unfolded state and have no biological activity. The formation of inclusion bodies is considered to be one of the major drawbacks of the pET system. To determine the ratio of the protein expressed by pET28 and pXWZ1 vector in the form of inclusion bodies, the QseB and GFPuv proteins were expressed and compared. Lanes 1 and 2 were supernatant and precipitation after ultrasonic fragmentation of GFPuv protein expressed by pET28(+) vector, respectively (ratio:1:1), and Lanes 3 and 4 were supernatant and precipitation after ultrasonic fragmentation of GFPuv protein expressed by pXWZ1 vector, respectively (ratio:1:4) (FIG. 4A). Likewise, as shown in FIG. 4B, Lanes 1 and 2 were supernatant and precipitation after ultrasonic fragmentation of QseB protein expressed by pET28(+) vector, respectively (ratio:2:1), and Lanes 3 and 4 were supernatant and precipitation after ultrasonic fragmentation of QseB protein expressed by pXWZ1 vector, respectively (ratio:1.5:1) (Fig. 4A). These results suggested that compared with the pET28(+) expression vector, the proportion of inclusion body produced by the pXWZ1 vector was significantly lower than the pET28(+) vector.

## Discussion

Bacteria use QS system to communicate with each other and synchronize behaviors as multicellular organisms. They secrete autoinducers to the environment and change the gene expression patterns once by detecting the concentration of these autoinducers reaching to a threshold. In this study, we utilized AI-2 QS system to realize the auto-induction of the heterogeneous protein expression in *E. coli*. It is interesting that only by inserting the *lsr* promoter into the pET28a(+) vector, the target genes expression was apparently higher than that expressed by the original pET28a(+) vector with induction of IPTG. As generally known, the host *E. coli* strain can produce LsrR protein which can also bind to the *lsr* promoter of the pXWZ1 vector to repress its downstream gene transcription. However, when the AI-2 secreted by the host *E. coli* accumulated to a threshold in the culture medium, the internalized and phosphorylated AI-2 can relieve the repression of the LsrR protein and initiate the target gene transcription (FIG. 1A). Therefore, the pXWZ1 vector can initiate target gene transcription without the T7 RNA polymerase.

In the process of heterogeneous proteins expression by the original pET28a(+) vector, the presence of IPTG is indispensable. However, addition of IPTG is not only toxic to the host strain, but also imposes a metabolic burden which leads to the enhanced formation of IBs. A careful balance between transcription and protein folding must be considered to reduce the amount of IBs. Therefore, a series of experimental conditions including pH of the culture medium, induction temperature, and the amino acid sequence of the product have to be changed and explored (Strandberg & Enfors 1991; Wurm et al., 2018). The IPTG induction method which is money-consuming and requires considerable effort waits to be improved. In this study, we modified the pET28a (+) vector and realized the autoinduction of this vector. We found that the target gene transcript strength by the new vector without any exogenous inducer was stronger than that by the original vector with the addition of IPTG. Compared with pET28a(+) protein expression vector, the obvious advantage of pXWZ1 expression vector is that it does not require IPTG to induce expression, has a high proportion of soluble proteins, and is more economical and convenient. However, it is uncertain whether the pXWZ1 expression vector can be widely used in *E. coli* hosts with non-inactivated *lsr* operon. Based on the current research results, it is of great significance that the application and optimization of the pXWZ1 expression vector will be further explored in future work.

Some previous investigations have also shown good results for diverse products gained *via* induction systems, using mixtures of glucose, glycerol and lactose (Blommel et al., 2007; Kittler et al., 2020; Tahara et al., 2021). However, induction of the *lac* operon by lactose requires the addition of a small amount of lactose in the cultured cells, and induction is ineffective with strains that lack either of lactose permease and β-galactosidase activities (Studier, 2005; Swigon, Coleman & Olson, 2006). Additionally, in the glucose-lactose induction system, the content of glucose and lactose needs a suitable ratio to promote induction. And the continuous fermentation process requires multiple additions of lactose, which increases the risk of bacterial contamination (Pang et al., 2020; Tahara et al., 2021). By contrast, because AI-2 is a signal molecule synthesized by E. coli itself, so the advantages of AI-2 auto-induction system are that no additional inducers need to be added in the continuous fermentation process, and the host selection is more extensive. In summary, this study provides a new perspective for the development and application of auto-induced expression. The pXWZ1 expression vector was characterized by reporter genes *gfpuv* and *lacZ*, which confirmed that the pXWZ1 expression vector has a practical application value. This new auto-induction system will exclude the limitations of IPTG induction and will become a more economical and convenient tool for the heterogeneous proteins expression in industry.

### Conclusions

In this study, the auto-inducible expression vector pXWZ1 was constructed by inserting the *lsr* promoter region into the pET28a (+) vector. The vector pXWZ1 was characterized by reporter genes *gfpuv* and *lacZ*, which confirmed that the pXWZ1can express protein without IPTG induction, and the soluble proteins account for a high proportion. This new auto-induction system resolves the limitations of the IPTG induction including toxic to host and increasing formation of inclusion body and will become a more economical and convenient tool for recombinant protein expression.

## Supplemental Information

10.7717/peerj.12497/supp-1 Supplemental Information 1 Promoter sequence of lsrA

10.7717/peerj.12497/supp-2 Supplemental Information 2 The data of Fluorescence intensity

10.7717/peerj.12497/supp-3 Supplemental Information 3 The data of β-galactosidase Activity

## Additional Information and Declarations

### Data Availability

The following information was supplied regarding data availability: The data of β-galactosidase activity, fluorescence intensity, and promoter sequence are available in the Supplementary Files.
