# Supplementary material for: Construction of an AI-2 quorum sensing induced heterologous protein expression system in Escherichia coli
Source: PeerJ. 2021 Nov 16;9:e12497. doi: 10.7717/peerj.12497 (PMC8603832; doi:10.7717/peerj.12497)
Supplement: Supplemental Information 1 [file peerj-09-12497-s001.docx]

**Promoter sequence of *lsrA***

**5’**AATTCATTCTTCACTTTGAACATATTTAAATCTTTAATGCAATTGTTCAGTTCTTGCTCATTTATATCTGTGATGGCAACCACAGTTTGACTCTACGAGCATGAACAAACGCAACCGTGAAAATCAAAATAGCATAAATTGTGATCTATTCGTCGGAAATATGTGCAATGTCCACCTAAGGTTATGAACAAATTAAAAGCAGAAATACATTTGTTCAAAACTCACCTGCAAAACTGAACGGGGGAAAT **3’**
